# A Topical Combination of Xyloglucan and Pea Protein Effectively Manages Oral Ulcers In Vivo

**DOI:** 10.1111/jop.70029

**Published:** 2025-08-11

**Authors:** Alessia Filippone, Giovanna Casili, Marika Lanza, Michela Campolo, Irene Paterniti, Laura Cucinotta, Rossella Basilotta, Alessio Ardizzone, Emanuela Esposito

**Affiliations:** ^1^ Department of Chemical, Biological, Pharmaceutical and Environmental Sciences University of Messina Messina Italy

**Keywords:** aphthous stomatitis, pea protein, xyloglucan

## Abstract

**Background:**

Aphthous stomatitis (AS) is a common idiopathic condition that affects approximately 5% to 25% of the general population. AS is characterized by recurrent painful aphthous ulcers on non‐keratinized oral mucous membranes. Lifestyle modifications and topical treatments are often used to manage symptoms, but no definitive cure exists. This study aims to evaluate the ability of a topical gel containing xyloglucan and pea protein (TXP), two mucoprotective substances, to restore the integrity of oral epithelial cells and manage inflammation associated with AS.

**Methods:**

In this study, oral ulcers were induced in rats by applying a phenol solution for 60 s to the mucosa of the left cheek. Following the formation of the lesion (24 h later) topical treatment with TXP was carried out for 2, 4, and 5 days. The healing properties of TXP were assessed by histopathological examination of the oral mucosa through the evaluation of morphological criteria such as hyperemia, edema, and presence of neutrophilic infiltration and by measuring the expression levels of inflammatory markers such as TNF‐α and IL‐2.

**Results:**

Results demonstrated that TXP was able to effectively treat oral ulcers by reducing the degree of hyperemia and edema in cheek tissue already at day 4. Moreover, TXP significantly decreased the levels of IL‐2 and TNF‐α after 4 days of gel application.

**Conclusions:**

This study demonstrates that TXP possesses a significant ulcer repairing ability in vivo, as it effectively restores the integrity of oral mucosal tissue, thus helping to reduce inflammation.

## 1 Introduction

Aphthous stomatitis (AS), or canker sores, is the most common inflammatory ulcerative condition of the oral cavity affecting the oral mucosal layer, tongue, and gums, with a global incidence of 5% to 25%. AS is characterized by recurrent painful aphthous ulcers on the non‐keratinized oral mucous membranes that can greatly impact the quality of life by interfering with functions such as talking, eating, and swallowing [1]. The cause of AS is idiopathic and multifactorial, and although not fully elucidated, it might involve activation of the cell‐mediated immune system, alterations of the oral microbiome, or hematinic deficiencies [2]. Moreover, several factors, including hormonal changes, local injury, drug use, food hypersensitivity, stress, and genetic inheritance, are known to contribute to the development of oral lesions in AS [3].

At the histopathological level, aphthous ulcers are characterized by epithelial oedemic lesions and infiltration of inflammatory mononuclear cells and neutrophils, which release pro‐inflammatory mediators. Furthermore, AS is known to cause anatomical changes in the oral epithelial tissue, including an imbalance of adherent junctions (particularly E‐cadherin), epithelial tight junctions (TJs) and mucin proteins, all of which play a crucial role in cell growth, differentiation, migration, and production of mucus [4, 5]. Such disruption of the epithelial barrier can expose the immune cells of the lamina propria to oral antigens, which could trigger the inflammatory response typical of AS. In worst cases, this could lead to tissue atrophy, hyperkeratosis, and/or acanthosis.

In this perspective, the management of AS largely depends on the severity and cause of the lesions [6, 7]. Common local treatments include antiseptics (e.g., chlorhexidine gluconate), antibiotics, local anesthetic gels, corticosteroids (e.g., prednisone), and topical anti‐inflammatory agents, while oral corticosteroids and systemic immunomodulators (e.g., thalidomide) are prescribed in case the patient does not respond to the topical therapy [8]. However, the efficacy of most local therapies is continuously affected by saliva secretion and oral muscle movements that limit the adhesion time of the products to the ulcer [9]. Moreover, long‐term use of some of these treatments is known to be associated with several side effects, mainly due to systemic absorption, such as headache and gastrointestinal symptoms, among others. In general, the current treatment approach aims to give symptomatic pain relief and reduce local inflammatory responses to accelerate the healing time, without effectively restoring the disrupted epithelial barrier.

In the last years, plant‐derived compounds have gained considerable interest thanks to their ability to manage mucosal dysfunction [10]. However, naturally derived safe and effective treatments with a clinically proven and fast action remain limited and highly desired by patients. In this perspective, xyloglucan (XG) and pea protein (PP) are emerging for their promising protective effects. XG is a polysaccharide contained in the seeds of the tamarind tree (*Tamarindus indica, L*.) with mucoprotective and mucoadhesive properties [11]. XG has been found to form a protective film on the human intestinal mucosa, allowing the restoration of the physiological function of intestinal epithelial cells. Moreover, XG showed exert protective actions by forming a barrier over diverse epithelial tissues under preclinical settings, such as a murine model of allergic rhinitis and preclinical models of urinary tract infections (UTIs) [12].

PP is derived from *Pisum sativum* (peas) and is known to be a good source of essential amino acids. In the past, XG and PP have been demonstrated to synergistically create a protective mechanical barrier over the intestinal epithelial cells in several in vivo models of gastrointestinal symptoms [13], where these ingredients were able to restore the physiological barrier property of the epithelia. Moreover, XG and PP were clinically effective in reducing symptoms of diarrhea‐predominant IBS patients [14, 15] as well as functional abdominal bloating and distension [16]. Similarly, this combination of ingredients applied topically was effective in reducing signs and symptoms of skin diseases such as atopic dermatitis and psoriasis in preclinical models [17, 18, 19], as well as in clinical practice [20, 21].

Based on this body of evidence, this study aims to determine the ability of a topical gel containing XG and PP (TXP) to restore the oral epithelial tissue integrity and control AS‐associated inflammation in a murine model of AS.

## 2 Materials and Methods

### 2.1 Materials

All chemicals were obtained from the highest grade of commercial sources. The product TXP/MD containing a combination of xyloglucan and pea protein was kindly provided by DEVINTEC SAGL (Lugano, Switzerland).

### 2.2 Animals

Male Wistar rats (weight, 180–220 g) (Harlan, Milan, Italy) (n tot = 72) were housed in a controlled environment (22°C± 2°C, 55% ± 15% relative humidity, 12 h light/dark cycle). After a one‐week acclimation, rats were fed a standard diet and water. The animals' care has been approved by the Board of Auditors of the University of Messina and complies with the regulations in Italy (DM 116192) and Europe (European Directive 2010/63/EU amended by Regulation 2019/1010). The animals used for this study were randomly selected from those suitable and available at that time.

### 2.3 Phenol‐ Induced Oral Ulcer Model

Oral ulcers were induced as previously described [22]. Briefly, a small cotton ball was placed at one end of a glass tube with a diameter of 3 mm, soaked in a solution of phenol and then placed on the left cheek mucosa of the animals for 60 s to induce the lesion. After 24 h, all rats developed ulcers of 3 mm in diameter on the oral mucosa. The tissues of oral ulcers (left cheek pouches) of each group were processed for morphological, histological, and biochemical analysis.

### 2.4 Experimental Groups

Wistar rats were randomly divided into six groups:

- Group 1 (Sham + saline): saline was applied to the cheek mucosa of rats (*n* = 12);
- Group 2 (Sham + TXP 5 days): TXP was applied for 2 days to the cheek mucosa of rats (*n* = 12);
- Group 3 (Sham + TXP 4 days): TXP was applied for 4 days to the cheek mucosa of rats (*n* = 12);
- Group 4 (Sham + TXP 2 days): TXP was applied for 5 days to the cheek mucosa of rats (*n* = 12);
- Group 5 (AS + saline): rats with oral ulcers sacrificed at day 5; saline was applied to the cheek mucosa (*n* = 12);
- Group 6 (AS + TXP), 2 days: rats with oral ulcers, TXP was applied to the cheek mucosa for 2 days (*n* = 12);
- Group 7 (AS + TXP), 4 days: rats with oral ulcers; TXP was applied on the cheek mucosa for 4 days (*n* = 12);
- Group 8 (AS + TXP), 5 days: rats with oral ulcers; TXP was applied on the cheek mucosa for 5 days (*n* = 12).

Rats in groups “AS + TXP, 2 days” and “AS + TXP, 4 days” were sacrificed respectively at day 2 and 4, while animals in the control groups and in “AS+TXP, 5 days” group were sacrificed at day 5. Data from mice belonging Groups 3 and 4 were not shown because we did not observed any significant changes in macroscopic signs, so we did not consider them for further analysis.

### 2.5 Sample Size Calculation

The minimum number of rats for every technique was estimated with the statistical test “ANOVA: Fixed effect, omnibus one‐way” with G‐power software. This statistical test generated a sample size equal to *n* = 12 rats for each technique and *n* = 24 for each group.

### 2.6 Macroscopical Analysis of Cheek Pouch

Macroscopic analysis, including detection of inflammatory features, such as erythema, erosion, vasodilatation, epithelial ulcerations, and abscesses, was assessed in cheek pouch mucosa as previously described [23]. The effects of TXP were evaluated macroscopically, based on the maximum diameter of ulcer tissue observed (< 1 mm, judgment healing, ≥ 1 mm, judgment not healing), on edema, and on the degree of hyperemia around ulcer. Scoring was defined as follows: I, hyperemia diameter < 1 mm, normal epithelium and connective tissue without vasodilatation, absence of or discreet cellular infiltration, and absence of haemorrhagic areas, ulcerations, or abscesses; score II, hyperemia diameter 1–2 mm, discreet vasodilatation or re‐epithelisation areas, discreet inflammatory infiltration with mononuclear prevalence, and absence of haemorrhagic areas, oedema, ulcerations, or abscesses; score III, hyperemia diameter 2–3 mm, moderate vasodilatation, areas of hydropic epithelial degeneration, inflammatory infiltration with neutrophil prevalence, presence of haemorrhagic areas, oedema, and eventual ulceration, and absence of abscesses; score IV, hyperemia diameter > 3 mm, severe vasodilatation and inflammatory infiltration with neutrophil.

### 2.7 Histological Analysis

The collected cheek pouch mucosa tissues were fixed with 10% neutral formalin, dehydrated with graduated ethanol, and embedded in paraffin. Subsequently, the 7 μm thick tissue sections were deparaffinized with xylene and stained with hematoxylin and eosin. All sections were evaluated using an AxioVision microscope (Zeiss, Milan, Italy) and the histological results were visualized at ×10 and ×20 magnification (100 μm and 50 μm of the scale bar, respectively). Inflammatory cell infiltration, vasodilatation, presence of hemorrhagic areas, edema, ulcerations, and abscesses were assessed in a single‐blinded fashion and graded, as previously described by Medeiros et al., 2011 [23]. The score used was the following: score 1, normal epithelium and connective tissue without vasodilatation, absence of or discreet cellular infiltration, and absence of hemorrhagic areas, ulcerations, or abscesses; score 2, discreet vasodilatation or re‐epithelization areas, discreet inflammatory infiltration with mononuclear prevalence, and absence of hemorrhagic areas, edema, ulcerations, or abscesses; score 3, moderate vasodilatation, areas of hydropic epithelial degeneration, inflammatory infiltration with neutrophil prevalence, presence of hemorrhagic areas, oedema, and eventual ulceration, and absence of abscesses; score 4, severe vasodilatation and inflammatory infiltration with neutrophils.

### 2.8 The Analysis of Tumor Necrosis Factor‐a (TNF‐a) and Interleukin‐2 (IL‐2) Cheek Pouch Mucosa Tissues

Cheek pouch mucosa tissues were collected and stored at −80°C until required. Collected tissue was homogenized and processed, and levels of TNF‐α and IL‐2 in the samples were determined with a commercial ELISA kit (R&D Systems, Minneapolis, MN, USA, #RAB0480 #CSB‐E04628r, respectively) as previously described [24]. Cytokine levels were evaluated by using UV–VIS spectrophotometry—absorbance measured at 490 nm.

### 2.9 Statistical Analysis

All values reported in the figures and in the text are indicated as mean ± standard deviation (SD) and are representative of at least three independent experiments. For data analysis, firstly we assessed the normality distribution through the Shapiro–Wilk test; then, according to the obtained results, we used the One‐Way ANOVA followed by a Bonferroni post hoc test for multiple comparisons or by the Kruskal‐Wallis test as for H&E and macroscopic evaluations. A *p*‐value of less than 0.05 was considered significant.

## 3 Results

### 3.1 Macroscopic Analysis of Cheek Pouch

After ulcer induction, the damaged oral mucous membrane of untreated groups (Group 5) appeared pitted and generally round, characterized by high hyperemia and edema. Interestingly, TXP applied for 5 days (Group 8 *p* < 0.05) significantly reduced the degree of hyperemia and edema compared to the control's groups (Groups 1–2–3 and 4). Instead, TXP topically applied only for 2 and 4 days (Groups 6 and 7) did not show a significant reduction in edema and mucosal degradation (Table 1).

**TABLE 1:** Macroscopic analysis of cheek pouch. In each experimental group the number of mice was *n* = 12.

Groups	N°	I	II	III	V	Mean ± SD	CI	[*p*]
Group 1 (Sham + veh)	12	12	0	0	0	1.00 ± 0.00	1.00–1.00	### (*p* < 0.001)
Group 2 (Sham + TXP 5 days)	12	12	0	0	0	1.00 ± 0.00	1.00–1.00	### (*p* < 0.001)
Group 3 (Sham + TXP 4 days)	12	12	0	0	0	1.00 ± 0.00	1.00–1.00	### (*p* < 0.001)
Group 4 (Sham + TXP 2 days)	12	12	0	0	0	1.00 ± 0.00	1.00–1.00	### (*p* < 0.001)
Group 5 (Phenol + veh, 5 days)	12	0	0	8	4	3.33 ± 0.49	3.02–3.65	*** (*p* < 0.001)
Group 6 (Phenol + TXP, 2 days)	12	0	3	4	5	3.15 ± 0.82	2.63–3.67	ns (*p* > 0.99)
Group 7 (Phenol + TXP, 4 days)	12	1	5	6	0	2.42 ± 0.67	1.99–2.84	ns (*p* = 0.81)
Group 8 (Phenol + TXP, 5 days)	12	3	5	4	0	2.02 ± 0.77	1.53–2.51	# (*p* = 0.04)

*Note*: Values are reported as means ± SD. Kruskal‐Wallis test. ***p < 0.001 vs. Sham + Veh; # *p* < 0.05 vs. Phenol + veh, 5 days (Group 5).

### 3.2 Histopathological Analysis of the Oral Mucosa

**FIGURE 1:**
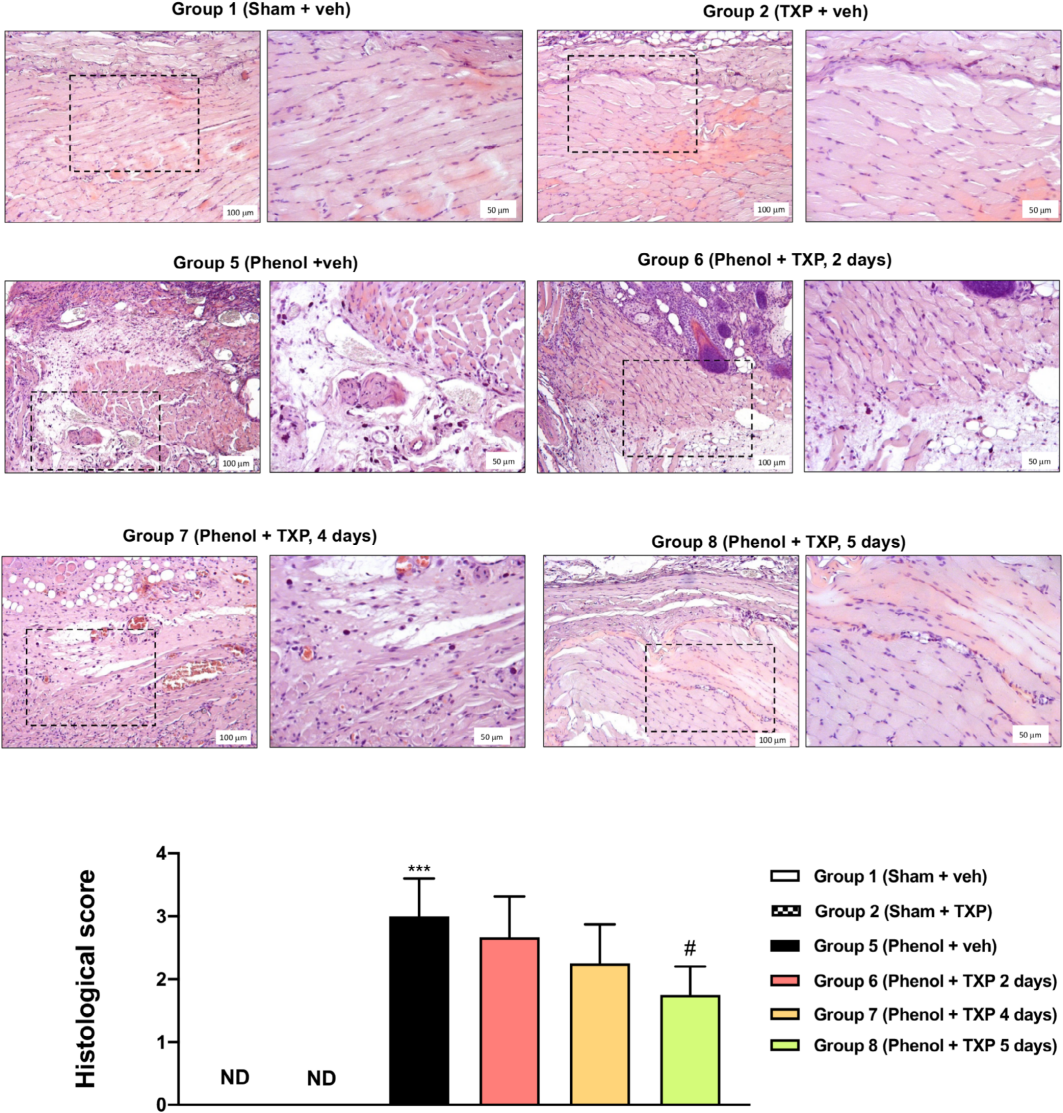
*Histopathological analysis of the oral mucosa*. No histological changes were found in oral mucosa tissue collected from control groups (Group 1 and Group 2), while the oral mucosa was destroyed and infiltrated by neutrophils in phenol‐induced rats (Group 5). TXP treatment for 5 days restored the histological alterations and reduced neutrophilic infiltration, while TXP treatment for 2 and 4 days did not show a statistically significant improvement in histological damage. In each experimental group, the number of mice was *n* = 12. Values are reported as means ± SD. Kruskal‐Wallis test. ***p < 0.001 vs. Sham + veh; # *p* < 0.05 vs. Phenol + veh, 5 days (Group 5); ND not detectable.

As showed in Figure 1, the oral mucosa and the squamous epithelium of untreated rats (Group 5) displayed significant damage, represented by the presence of necrotic tissue and infiltration of inflammatory cells (*p* < 0.001) compared to normal mucosa (Group 1). However, the degree of lesion as well as inflammatory cells significantly decreased in animals treated with TXP for 5 days (Group 8; *p* < 0.05) compared to the untreated control (Group 5). On the contrary, animals treated with TXP for 2 and 4 days (Group 6 and Group 7) did not display a significant reduction in oral ulcer damage at the microscopic level.

Raw data regarding histological analysis are provided in Table S1.

### 3.3 Analysis of Cytokines Production

**FIGURE 2:**
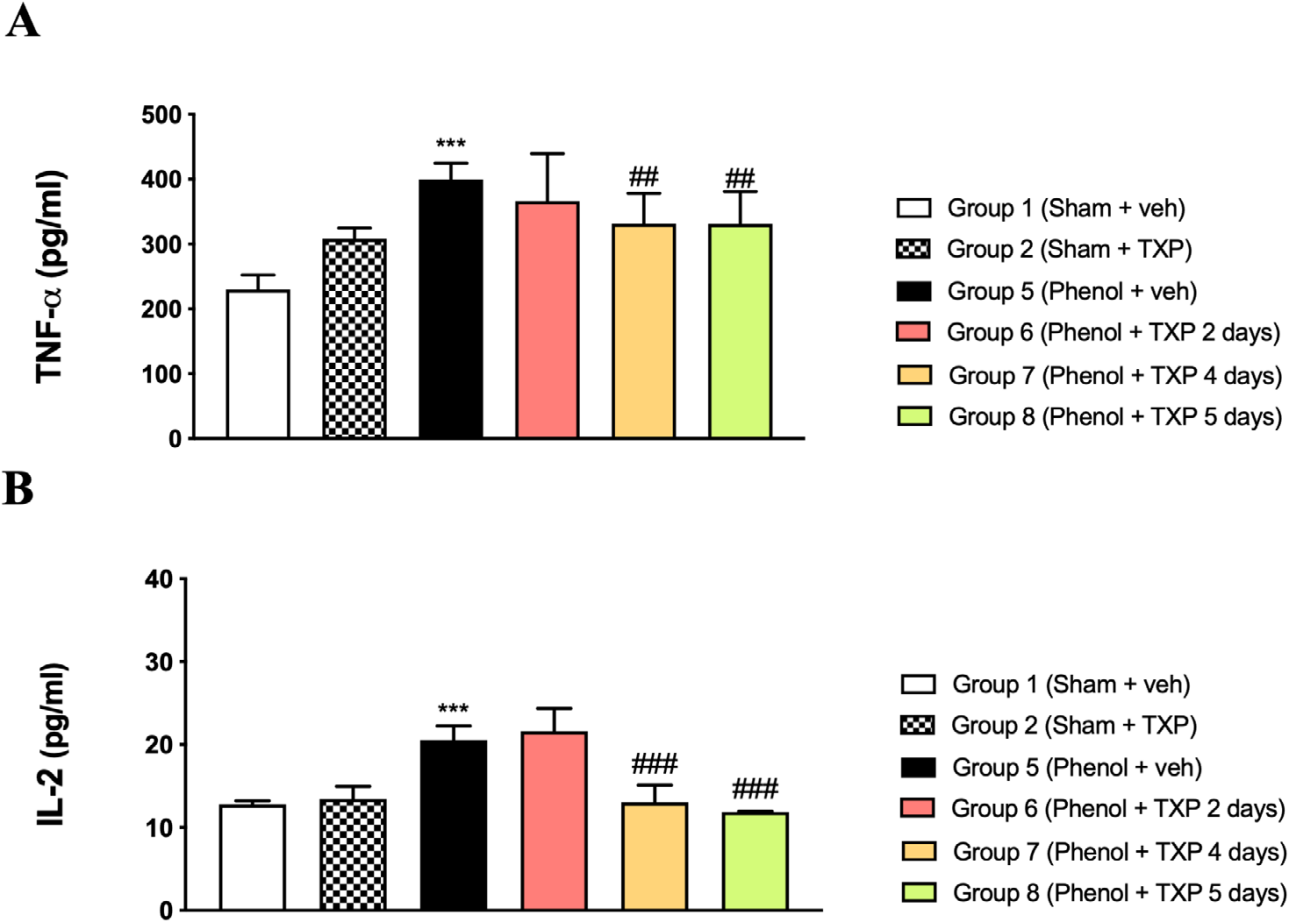
*Analysis of cytokine production*. The levels of TNF‐α were significantly increased in phenol‐induced rats (Group 5) compared to controls (Group 1 and Group 2) (A). This was significantly reduced by treatment with TXP for 4 and 5 days, but not by TXP treatment for 2 days (A). The levels of IL‐2 were significantly increased in phenol‐induced rats (Group 5), compared to controls (Group 1 and Group 2) (B). Treatment with TXP for 4 and 5 days significantly reduced IL‐2 levels, while treatment with TXP for 2 days was insufficient to induce a significant reduction (B). ***p < 0.001 vs. Sham + veh; ## *p* < 0.01 vs. Phenol + veh, 5 days; ### *p* < 0.001 vs. Phenol + veh, 5 days.

Oral ulcers are often characterized by infiltration of inflammatory cells [25]. Immune cell activation, orchestrated by mucosal keratinocytes, affects endothelial cell adhesion and neutrophil chemotaxis. The crosstalk between immune and epithelial cells is mediated by cytokines, in particular TNF‐α and IL‐2. Here, TNF‐α and IL‐2 quantification was assessed in oral mucosa tissue. As expected, cytokine levels were increased in untreated groups (Group 5 *p* < 0.001) compared to normal mucosa (Group 1) (Figure 2A,B respectively). Interestingly, treatment with TXP for 4 days (Group 7) and 5 days (Group 8) significantly reduced both TNF‐α (*p* < 0.01 at day 4 and 5) and IL‐2 expression (*p* < 0.001 at day 4 and 5) (Figure 2A,B respectively), suggesting that TXP exerts a protective effect on oral mucosa. Contrarily, TXP administered for 2 days (Group 6) was not able to significantly reduce IL‐2 and TNF‐α levels (Figure 2A,B respectively).

Raw data regarding ELISA kits are presented in Table S1.

## 4 Discussion

In the oral cavity, the epithelium acts as a first line of defense against potential external harmful insults. In AS, loss of this physiological barrier function might imply alteration of TJs structure and subsequent infiltration of inflammatory molecules. Currently, pharmacological approaches for AS involve the use of topical products that reduce the symptoms, but do not actively promote mucosal reepithelization.

Natural extracts are becoming increasingly important in the treatment of AS. These compounds contain several types of secondary metabolites such as flavonoids, polyphenols, and water‐soluble lipophilic polysaccharides that possess anti‐inflammatory and soothing properties and can help reduce inflammation and pain associated with aphthous ulcers, providing immediate relief [26]. These bioactive ingredients have been reported to be primarily associated with non‐stick and anti‐inflammatory effects due to their stimulation of the immune response. Many substances, including *Chamomilla Recutita* , have been shown to accelerate the healing process of ulcerative lesions in in vivo models, representing a more tolerated alternative to corticosteroids [27]. Even *Copaiba oleoresin* and *Curcumin* have attracted attention for their potential therapeutic benefits in the treatment of oral ulcers, demonstrating powerful anti‐inflammatory effects and promoting rapid and complete re‐epithelialization of oral wounds [28, 29].

Therefore, it is necessary to identify safe and effective therapeutic strategies to quickly reduce the symptoms while restoring the oral physiological barrier, thus preventing AS recurrence. Previous studies have shown that XG and PP possess the ability to create a mechanical barrier over epithelial tissues and indirectly modulate the degree of inflammation, restoring the physiological homeostasis of tissues [13]. XG has been found to form a protective film on the human intestinal mucosa that acts as a mechanical barrier, protecting the underlying tissue from external irritants and pathogens and allowing the restoration of the physiological function of epithelial cells [30]. XG also exerts hygroscopic properties, which means it can retain moisture, keeping the mucosal surface hydrated and helping soothe the inflamed tissue and promote healing [31]. Although PP is primarily known for its nutritional value, it also possesses properties that could be beneficial in the management of oral mucositis. In fact, the high content of essential amino acids, including arginine, glutamine, and branched‐chain amino acids (BCAAs), is essential for the repair and regeneration processes of damaged mucosal tissues and for the promotion of anti‐inflammatory and antioxidant effects, which could help to reduce the severity of mucositis and support the healing process [32, 33]. Similar to XG, PP also possesses film‐forming capacity by building a protective barrier on the mucosal surface, which could help protect damaged areas from further irritation and maintain moisture in the mucosa [34, 35].

In the case of the topical treatment of oral mucositis, the duration of the protective effect of XG and PP is generally more relevant than its half‐life. In fact, their effectiveness is often linked to how long they manage to remain adherent to the surface of the oral mucosa [36]. The adhesion time of these compounds to the mucosa is influenced by saliva production, enzymatic activity, and mechanical forces of the oral environment, which favor their rapid metabolization by reducing the half‐life [37]. For this reason, one strategy to improve treatment efficacy is to use more complex delivery systems such as gel, hydrogel, or nanoparticle formulations [38, 39].

In this study, TXP gel formulation demonstrated a protective effect on the oral mucosa affected by AS by promoting the physiological mucosal integrity and improving tissue architecture; consequently, it reduced pro‐inflammatory cytokine levels.

The phenol‐induced oral ulcer is a widely used experimental model to reproduce mechanical AS‐like lesions in vivo, inducing an ulceration of the cheek pouch mucosa [22]. AS lesions are characterized by epithelial erythema, hyperaemia, haemorrhagic areas, and extensive ulceration, which, if not adequately treated, might lead to an intense inflammatory state with an increase in neutrophil infiltration, oedema, and abscesses. In this study, TXP treatment for 5 days considerably reduced the macroscopic damage induced by phenol, as treated animals displayed a lower degree of erythema and vasodilation, with no ulceration or hyperaemia [22]. Furthermore, histopathological examination revealed that at day 5 the microscopical damage was significantly reduced by TXP application, suggesting that TXP acted as a protective layer, promoting tissue reepithelization and avoiding infiltration of inflammatory cells.

It is well known that cytokines such as TNF‐α and IL‐2 play a crucial role in the development of oral ulcers in AS patients, as their overexpression has been reported in lesioned mucosa of AS patients [40, 41, 42]. In fact, immunomodulatory drugs have demonstrated to be effective in managing AS by inhibiting cytokine activity and/or production [43, 44]. In the present study, induction with phenol led to increased TNF‐α and IL‐2 levels in the oral tissues in all animals, while the use of TXP for 4 and 5 days significantly reduced the cytokine levels.

Taken together, these results support the hypothesis that the combination of XG and PP can be beneficial in the management of AS. Despite the promising preliminary results obtained, limitations of the study must be addressed. Considering rat and human oral mucosa have different gross anatomical structures, it is challenging to develop animal models that can fully reproduce the disease in humans. Therefore, future in‐depth clinical studies are needed to fully validate the efficacy of TXP in reducing pain and inflammation in AS patients, as well as its rapidity of action. Moreover, since TXP showed to be able to promote tissue integrity, its potential in prolonging the duration of ulcer‐free periods in patients with recurrent AS should be investigated. Nevertheless, TXP was able to reduce AS microscopical and inflammatory features in vivo, demonstrating that the protection of the oral epithelium in case of AS is an important target for an effective management of the disease.

## 5 Conclusions

The present study demonstrates the ability of TXP to efficaciously promote the healing of oral ulcers by reducing the degree of hyperaemia and oedema in cheek tissue, as well as significantly decreasing the levels of inflammatory cytokines. Therefore, TXP may represent a safe and effective alternative treatment for the management of AS.

## 6 Limitations of the Study

This study conducted on rats to test topical treatment with TXP for AS provides valuable information on its benefits in terms of anti‐inflammatory and protective activity of the oral mucous membranes but has some limitations. Anatomical differences in the oral structures of rats compared to those of humans could affect the absorption and efficacy of the gel. Therefore, it may be useful to consider comparing different types of drug formulations and delivery to increase the adhesion time of the gel in the oral mucosa, for example by considering mucoadhesive formulations. Therefore, although further human clinical trials are needed to confirm the efficacy and safety of the gel, the results of this study may offer initial insights into the beneficial effect of this compound in the management of AS.

## Author Contributions

**Michela Campolo:** conceptualization. **Irene Paterniti:** methodology. **Laura Cucinotta and Rossella Basilotta:** formal analysis. **Alessia Filippone:** investigation. **Marika Lanza:** resources. **Alessio Ardizzone:** data curation. **Giovanna Casili:** writing – original draft preparation. **Alessia Filippone:** writing – review and editing. **Michela Campolo:** visualization. **Irene Paterniti:** supervision. **Emanuela Esposito:** project administration. **Emanuela Esposito:** funding acquisition. All authors have read and agreed to the published version of the manuscript.

## Ethics Statement

The study was approved by OPBA of Messina with the authorization number 294/2021‐PR.

## Conflicts of Interest

The authors declare no conflicts of interest.

## Supporting information


**Table S1:** Descriptive statistics. Means ± SD of results obtained in the histological analysis and in TNF‐α and IL‐2 ELISA kits.

## Data Availability

All data in this study are included in this published article.
