# Supplementary material for: A Topical Combination of Xyloglucan and Pea Protein Effectively Manages Oral Ulcers In Vivo
Source: J Oral Pathol Med. 2025 Aug 11;54(9):790–7. doi: 10.1111/jop.70029 (PMC12521072; doi:10.1111/jop.70029)
Supplement: Supplementary file 1 — Table S1: Descriptive statistics. Means ± SD of results obtained in the histological analysis and in TNF‐α and IL‐2 ELISA kits. [file JOP-54-790-s001.docx]

**Supplementary Table 1**

|  | **Mean ± SD** | | |
| --- | --- | --- | --- |
| **Groups** | **Histological score** | **TNFα** | **IL-2** |
| **Group 1 (Sham + veh)** | 0.00 ± 0.00 | 230.30 ± 22.20 | 12.77 ± 0.46 |
| **Group 2 (Sham +TXP 5 days)** | 0.00 ± 0.00 | 308.30 ± 16.63 | 13.46 ± 1.48 |
| **Group 5 (Phenol + veh, 5 days)** | 3.00 ± 0.60 | 400.00 ± 24.79 | 20.54 ± 1.71 |
| **Group 6 (Phenol + TXP, 2 days)** | 2.67 ± 0.65 | 366.20 ± 72.94 | 21.57 ± 2.74 |
| **Group 7 (Phenol + TXP, 4 days)** | 2.25 ± 0.62 | 331.50 ± 46.79 | 13.06 ± 2.04 |
| **Group 8 (Phenol + TXP, 5 days)** | 1.75 ± 0.45 | 331.20 ± 49.71 | 11.88 ± 0.08 |
